# EBV persistence in gastric cancer cases conventionally classified as EBER-ISH negative

**DOI:** 10.1186/s13027-022-00469-5

**Published:** 2022-11-17

**Authors:** M. C. Siciliano, S. Tornambè, G. Cevenini, E. Sorrentino, M. Granai, G. Giovannoni, D. Marrelli, I. Biviano, F. Roviello, H. Yoshiyama, L. Leoncini, S. Lazzi, L. Mundo

**Affiliations:** 1 Section of Pathology, Department of Medical Biotechnology, University of Siena, Siena, Italy; 2 Department of Medical Biotechnology, University of Siena, Siena, Italy; 3 Institut Für Pathologie und Neuropathologie Abt. Allgemeine und Molekulare Pathologie und Pathologische Anatomie University of Tubingen, Tübingen, Germany; 4 Department of Human Pathology and Oncology, Surgical Oncology, Siena University, Siena, Italy; 5 Gastroenterology Unit, A.O.U.S. Policlinico S. Maria alle Scotte, Siena, Italy; 6 Department of Microbiology, Shimane University Faculty of Medicine, Izumo, Shimane Japan; 7 Health Research Institute, University of Limerick, Limerick, V94 T9PX Ireland

**Keywords:** Gastric adenocarcinoma, Gastric cancer with lymphoid stroma, Epstein Barr Virus, *Hit and run* mechanism

## Abstract

**Background:**

The Epstein-Barr virus (EBV) causes various B-cell lymphomas and epithelial malignancies, including gastric cancer (GC) at frequencies ranging from 5 to 10% in adenocarcinomas (ADK) to 80% in GC with lymphoid stroma (GCLS). Using high-sensitivity methods, we recently detected EBV traces in a large cohort of EBV-negative B-cell lymphomas, suggesting a *hit-and-run* mechanism.

**Methods:**

Here, we used routine and higher-sensitivity methods [droplet digital PCR (ddPCR) for EBV segments on microdissected tumour cells and RNAscope for *EBNA1* mRNA] to assess EBV infection in a cohort of 40 GCs (28 ADK and 12 GCLS).

**Results:**

ddPCR documented the presence of EBV nucleic acids in rare tumour cells of several cases conventionally classified as EBV-negative (ADK, 8/26; GCLS, 6/7). Similarly, RNAscope confirmed EBNA1 expression in rare tumour cells (ADK, 4/26; GCLS, 3/7). Finally, since EBV induces epigenetic changes that are heritable and retained after complete loss of the virus from the host cell, we studied the methylation pattern of EBV-specifically methylated genes (*Timp2*, *Eya1*) as a mark of previous EBV infection. Cases with EBV traces showed a considerable level of methylation in *Timp2* and *Eya1* genes that was similar to that observed in EBER-ISH positive cases and greater than cases not featuring any EBV traces.

**Conclusions:**

These findings suggest that: (a) EBV may contribute to gastric pathogenesis more widely than currently acknowledged and (b) indicate the methylation changes as a mechanistic framework for how EBV can act in a *hit-and-run* manner. Finally, we found that the viral state was of prognostic significance in univariate and multivariate analyses.

## Introduction

The Epstein-Barr virus (EBV) is a gamma-herpes virus that infects more than 95% of healthy adults worldwide. While most people carry EBV as a life-long asymptomatic infection, in some people, the virus is associated with a number of B cell and epithelial cell malignancies, including Burkitt’s Lymphoma (BL), nasopharyngeal carcinoma (NPC) and gastric cancer (GC) [1, 2]. In order to establish a lifelong carrier state, the virus expresses a set of viral proteins as well as viral miRNAs that may impact key cellular pathways and host cell homeostasis [3–5]. EBV is also able to elude the immune response [6–8] and to manipulate the host epigenetic machinery, resulting in long-lasting host epigenetic reprogramming [9–11]. However, its possible contribution to the pathogenesis of EBV-associated diseases is largely unknown.

The role of EBV is further confounded by the less than total association of the virus with histologically and molecular similar tumours. Indeed, it has been proposed that the virus may be responsible also for tumours diagnosed as EBV negative by a mechanism of *hit-and-run* [12, 13].This theory suggested that EBV can mediate cellular transformation (hit) through its viral proteins and being progressively lost (run) from the host cell after inducing heritable changes in the cellular genes [14].The loss of EBV genome occurs when there is no selective pressure for its maintenance, and it is mainly due to the known imperfect duplication and asymmetric partitioning of EBV episomes during S-phase and M-phase respectively [15, 16]. In the meantime, the acquisition of somatic mutations in cellular oncogenes/tumor suppressor genes [17, 18] will functionally compensate for the loss of EBV genome.

Support for this idea comes from anecdotal case report of NPC and BL primary tumours and cell lines which after several cell cycles spontaneously lost the viral episomal genome [19–22]. Furthermore, fragments of the EBV genome in single cells have been demonstrated in a series of Hodgkin and non-Hodgkin lymphomas and cell lines that by standard criteria (EBER in situ hybridization or EBNA1 immunohistochemistry) would be classified as virus-negative [12, 23].

These findings prompted us to investigate the presence of EBV traces in other EBV-linked malignancies where the virus seems to play an important role in the development and progression of the tumour, such as GC.

The percentage of EBV infection associated with GC tumours is highly variable [24]. Gastric cancer with lymphoid stroma (GCLS), also known as medullary or lymphoepithelioma-like GC (LELC), is a subset of GC known to harbour the EBV genome in a range varying from 22.5 to 100% of cases, in respect to the ordinary-type adenocarcinomas (ADK) cases where the virus is detected in 5–10% of tumours [25]. In The Cancer Genome Atlas (TCGA) project, GC is classified according to its molecular biology into EBV-associated GC (EBVaGC), microsatellite instable tumours (MSI), genomically stable (GS), and chromosomally instable (CIN) [26]. Although the impact of EBV is still comprehensively unknown, several papers suggest that the virus contributes to GC tumourigenesis by inducing epigenetic changes [27, 28].

In fact, EBV-positive tumours show a higher prevalence of DNA hypermethylation than the other GC subtypes. Matsusaka and colleagues, using Illumina’s Infinium Bead Array, identified three epigenotypes groups based on the EBV status and methylation degree: (1) genes specifically methylated in the EBVaGC (such as *Timp2*), (2) genes methylated both in EBV positive and EBV negative/high tumours (such as *Eya1*) and, (3) genes methylated in all gastric cancers [29, 30]. Both TIMP2 and EYA1 proteins have been identified as tumour promoters in various cancers inducing cell migration and tumour metastasis [31, 32]. In addition, all EBV-positive tumours display amplification of *JAK2, CD274* (also known as *PD-L1*) and *PDCD1LG2* (also named *PD-L2*) and carry a lower number of mutations in genes such as *TP53, CDH1, RHOA*, than the other GC subgroups where these genes are found to be highly mutated [26, 33]. This pattern of mutations seems to mirror what has been observed in EBV-associated lymphomas such as BL and Hodgkin lymphoma (HL), where a *hit-and-run* mechanism has been proposed [23, 34].

The aim of this work was to investigate the presence of EBV traces also in GCs conventionally classified as EBV-negative by applying highly sensitive methods for EBV detection such as droplet digital PCR (ddPCR) for conserved EBV genomic regions and RNAscope for EBNA1 mRNA in situ detection [15, 35]. In addition, we studied the methylation pattern of genes specifically methylated by the virus (e.g., *Timp2*) as indirect proof of the *hit-and-run* mechanism. Finally, we investigated the impact of EBV presence (EBERs or EBV traces) on the overall survival (OS) of all patients.

## Materials and methods

### Cases selection

**Fig. 1:**
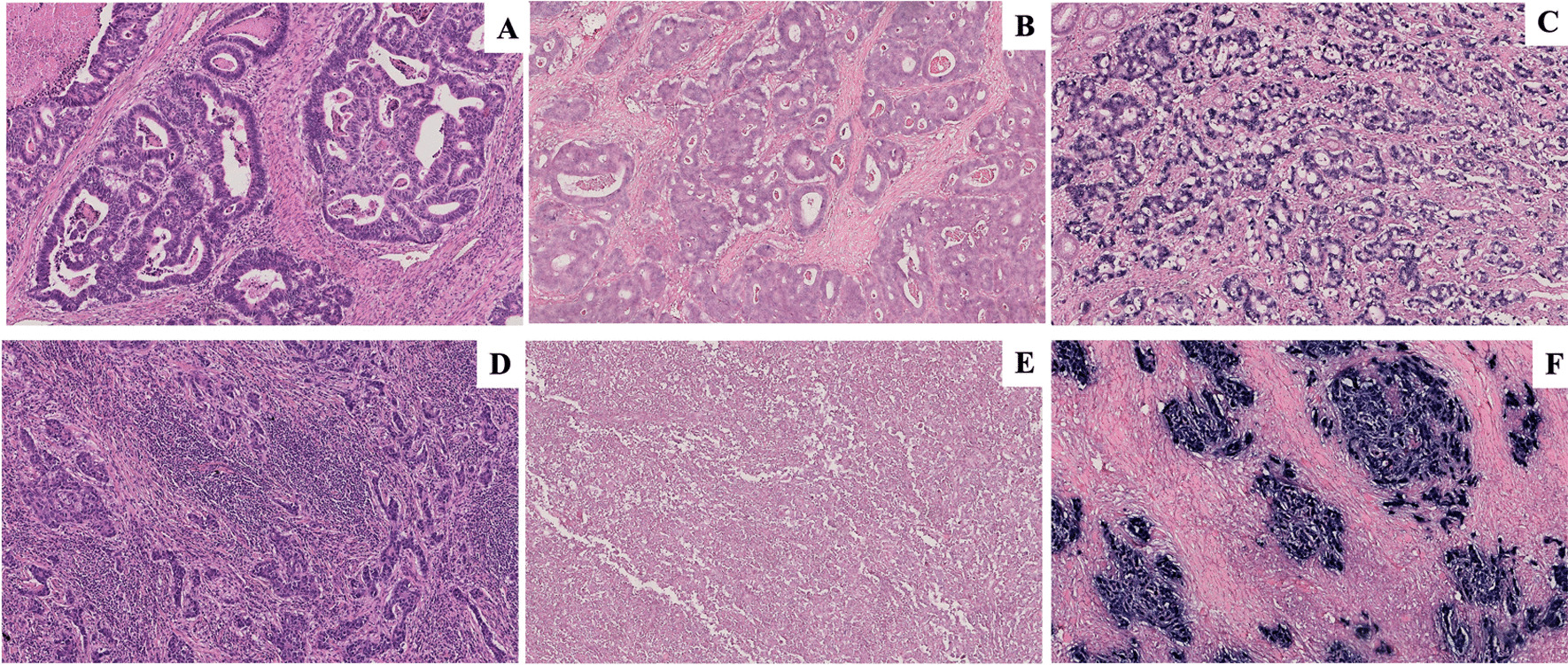
Histological and in situ hybridization findings. (**A**–**C**) Gastric adenocarcinoma; the tumor is composed of dilated or branching tubules invading the muscle layer HE (**A**), EBER-ISH negative (**B**), EBER-ISH positive; EBV-encoded small ribonucleic acid (EBER1) in situ hybridization shows positive nuclei in the neoplastic glands whereas the stroma remain unlabeled (**C**); (**D**–**F**) Gastric carcinoma with lymphoid stroma; cancer cells forming small nests or fused glands, accompanied by abundant lymphocyte infiltration HE (**D**), EBER-ISH negative (**E**), EBER-ISH positive; nuclei of carcinoma cells are positive by in situ hybridization targeting EBER, but the infiltrating lymphocytes are negative (**F**). (Original magnification 10X)

7 EBER-ISH positive GCs, selected as *gold standard* cases, were compared to 33 EBER-ISH negative consecutive GC observed at the Department of Surgical Oncology of Siena University Hospital in the year 2010 to allow a large prospective cohort study based on a significant long follow up. Histologic diagnosis was determined in accordance with the World Health Organization (WHO) criteria for gastric tumours. Among 7 EBER-ISH positive GCs, 5 were classified as GCLS and 2 as ADK. The remaining 33 EBER-ISH negative consecutive GC samples were grouped as 26 ADK and 7 GCLS (Fig. 1A–D). All the procedures were carried out automatically on representative paraffin sections from each case by Bench Mark Ultra (Ventana, Monza, Italy). The clinicopathologic features of cases consisting of age, gender and site were reported in Table 1.

**Table 1:** Clinicopathological features of 40 GC samples characterized by age, gender and site

Case Number	Age	Gender	Site	Histological Type
#1	78	M	Lesser curvature	ADK
#2	52	M	Lesser curvature	
#3	80	M	Lesser curvature	
#4	84	M	Lesser curvature	
#5	77	M	Pylorus	
#6	81	M	Pylorus	
#7	74	M	Cardia	
#8	58	M	Antrum, pylorus	
#9	84	M	Lesser curvature	
#10	70	F	Antrum	
#11	73	F	Antrum	
#12	63	M	Antrum, pylorus	
#13	63	M	Body of stomach, fundus	
#14	50	M	Cardia	
#15	90	F	Body of stomach, antrum	
#16	76	M	Antrum	
#17	80	M	Lesser curvature	
#18	79	M	Body of stomach, antrum	
#19	80	F	Lesser curvature	
#20	71	F	Greater curvature	
#21	89	F	Lesser curvature	
#22	89	F	Pylorus, antrum,body of stomach	
#23	85	M	Angulus, lesser curvature	
#24	63	M	Lesser curvature	
#25	69	M	Cardia, antrum	
#26	70	M	Antrum	
#27	64	M	Antrum	
#28	61	M	Antrum	
#29	59	F	Angulus	GCLS
#30	75	F	Antrum, lesser curvature	
#31	78	F	Antrum	
#32	61	F	Body of stomach	
#33	92	M	Lesser curvature	
#34	82	M	Cardia	
#35	76	F	Antrum	
#36	72	M	Cardia, body of stomach, Antrum	
#37	83	M	Fundus	
#38	78	F	Cardia	
#39	46	M	Body of stomach, lesser Curvature	
#40	69	F	Body of stomach, antrum	

### EBER-in situ hybridization

To validate the presence of EBV, EBER-ISH staining was performed on all 5 μm FFPE cases by an automated staining system (Ventana BenchMark ULTRA, Roche diagnostic, Monza-Italy), as previously described [18]. All steps were performed inside the instrument, from deparaffinization to counterstaining with appropriate positive and negative controls included in each staining run. A control slide prepared from a paraffin-embedded tissue block containing EBV-positive metastatic nasopharyngeal carcinoma in a lymph node accompanied each hybridization run. The EBER-ISH stained sections were scanned and analysed by Hamamatsu NanoZoomer-XR digital whole slide scanner.

### Laser capture microdissection (LCM)

The neoplastic cells of each GC sample (including those positive at EBER-ISH assay) were microdissected from EBER-ISH stained 3 μM-thick FFPE sections using a PixCell II laser capture microdissector (Arcturus Engineering, MGW, Florence, Italy). Multiple areas, each one containing ~ 40–50 cells, were harvested to collect a total number of ~ 200,000 cells. The EBER-ISH staining allowed us, in cases with EBER-negative cells, to exclude by microdissection even a small single EBER-positive reactive lymphocyte that might impact the following ddPCR analyses.

### DNA extraction and processing before droplet digital PCR

DNA was extracted from FFPE of the original neoplastic samples using NucleoSpin Tissue (Machery-Nagel, Italy) following manufacturer’s instructions. Positive and negative controls in addition to a blank control (H_2_O) were included in all runs. The amount and quality of DNA were evaluated by measuring the optic density (OD) at 260 nm, the 260/230 and the 260/280 ratios using a Nanodrop spectrophotometer (ND-100, Nanodrop, Thermo Scientific, Celbio, Italy).

### Droplet digital PCR assay to measure the absolute copy number of EBV genome load

ddPCR was performed using 200 ng of DNA, 1 × ddPCR Supermix for Probes (BioRad, Hercules, CA, USA), 900 nM of each primer, and 250 nM of the probe in a total volume of 22 µL, as previously described [23]. Among the fragments of the EBV genome, we choose to target in our cases BamHI-W and EBNA1, the most conserved region of EBV genome [36, 37]. BamHI-W is a reiterated sequence present at approximately ten copies per EBV genome and appears to be the most sensitive method to prove the presence of the viral genome, whereas EBNA1 probe targets a single-copy highly conserved gene, essential for maintaining the virus long-term in dividing cells. The absolute copy number of each viral assays was calculated by Bio-Rad software and showed as number of copies/µl. As previously reported, we used as biological negative controls a sample of Hair Cell Leukemia (HCL), whom has never been associated with EBV. In addition, we used water as technical negative controls for the ddPCR amplification (data not shown) [23].

### In situ detection of EBNA1 mRNA by RNAscope assay

RNAscope is an amplified ISH assay more sensitive than standard ISH for EBV-encoded RNAs to detect viral gene expression. It employs a multiple probe pair design strategy in which two independent probes within each pair (double Z probes) have to hybridize to the target sequence in tandem next to each other for signal amplification to occur, which improves the signal-to-noise ratio. Signal amplification is achieved by a cascade of hybridization of a nucleic acid pre-amplifier followed by multiple amplifiers that serve as a substrate for the subsequent binding of chromogenic molecules to the numerous binding sites in each amplifier. RNAscope is made to work for optimal RNA detection. The RNAscope workflow does not have any DNA-specific denaturing steps that will hinder RNA probe binding.

RNA in situ hybridization was performed using the RNAscope 2.0 HD Red Chromogenic Reagent Kit (Advanced Cell Diagnostic, CA), V-EBV-EBNA1 and V-EBV-EBER1 target probe, according to the manufacturer’s instructions. Each sample was quality-controlled for RNA integrity with a probe specific to the housekeeping *PPIB* mRNA used as positive control. Signal detection was performed by hybridizing the FastRed probe mix (RED-A and RED-B) on each sample and counterstaining the section with haematoxylin. RNA staining signals were identified as red punctate dots also visible by optical microscope. RNAscope scores were obtained independently by two pathologists (SL, LL) by counting the red dots per cell in ten high magnification field (40X).

Background staining was evaluated using a negative control probe specific for bacterial dihydrodipicolinate reductase (*dapB)*; all gastric cancer cases analysed did not show any dots for *dapB* in any cells (data not shown) [35]. RNAse (Qiagen, Hilden, Germay) pre-treatment, at 5 mg/mL final concentration in 1X PBS, was performed to demonstrate lack of signal after RNAScope protease digestion step.

### RNAscope and immunofluorescence (IF) staining for the simultaneous detection of EBNA1 mRNA detection and pan-keratin marker

Since EBNA1 mRNA signals detected by RNAscope can be visualized by an epifluorescence microscope, to further confirm the presence of EBNA1 mRNA in neoplastic cells, we performed a PanKeratin immunofluorescence on the same EBNA1 mRNA RNAscope stained section. Briefly, after deparaffinization and rehydration, heat-induced antigen retrieval was performed using a citrate-based solution (pH 6.0). We investigated the presence of Pan-Keratin using the AE1/AE3/PCK26 antibody (Ventana, 760–2595, Tucson, USA) and the fluorophore-conjugated secondary antibody FITC (MerckMillipore, AP124F, Darmstadt, Germany). The colour assignment and staining location is green-membranous. In order to detect nuclei signals, 40,6-diamidino-2-phenylindole (DAPI, ProLong™ Diamond Antifade Mountant with DAPI, P36966, Invitrogen, Country) was added to the slides. Tissue sections from the same set of cases and without antibody/fluorophore were used as negative control. The acquisition of multiplex IF staining reaction were performed using Tissue FAXSFluo slide scanning system (TissueGnostics, Vienna, Austria) based on a Zeiss Axio Imager Z2 upright epifluorescence microscope.

### Methylation studies

Genomic DNA was extracted from five 5-μm-thick whole sections of FFPE gastric cancer sections using the NucleoSpin Tissue extraction kit (Macherey–Nagel, Germany) according to the manufacturer’s instructions. The amount and quality of DNA were evaluated by measuring the optical density (OD) at 260 nm, the 260/230 and the 260/280 ratios using a Nanodrop spectrophotometer (NanoDrop Technologies LLT, USA). 300 ng of DNA from each sample were used for bisulfite conversion, as previously described [23]. Unmethylated cytosine was converted to uracil with the EpiTect Fast DNA bisulfite kit (Qiagen, Hilden, Germany, 59,824) according to the manufacturer’s instructions. Human HCT116 DKO Non-methylated and Methylated DNA (Zymo Research, USA, D5014-1/2) were used as standard controls. The signal and target CpGs were evaluated by using the PyroMark Q96 ID System (Qiagen, Hilden, Germany) which converts the pyrograms to numerical values for peak heights and calculates the proportion of methylation at each base as a C/T ratio. Along with standards, a cytosine not followed by a guanine, which was not methylated, served as an internal control to verify the efficiency of bisulfite conversion.

### Statistical analysis

Kaplan–Meier curves for the three viral states were drawn and statistically compared pairwise by means of the log rank test. The individual prognostic power (univariate analysis) of the various clinicopathological factors, in relation to survival time, was statistically assessed by Cox survival regression. The identification of a multivariate model explaining survival by combinations of statistically significant prognostic factors was also investigated, while still using Cox survival analysis. For all statistical analyses, the significance level was set at 95% (*p* ≤ 0.05).

## Results

### Findings at in situ hybridization (ISH)

The presence of EBV was detected in 2/28 (7%) ADK and in 5/12 (42%) GCLS samples (Fig. 1C–F) whereas the remaining cases were negative (Fig. 1B–E). The EBER-ISH positive case showed a distinctive diffuse nuclear stain. The intensity varied slightly from tumour to tumour but was consistent within the same tumour. No relationship was found between the intensity of EBER-1 expression and any clinicopathological features.

### Viral genome load by droplet digital PCR in GC tumours

The neoplastic cells of each GC sample (including those positive at EBER-ISH) were microdissected from EBER-ISH stained FFPE sections and screened by ddPCR to independently measure the absolute copy numbers of EBNA1 and BAMH1-W fragments. As expected, ddPCR analysis of the EBER-ISH positive cases (2 ADK and 5 GCLS) revealed high viral loads of EBNA1 (ADK, EBNA1: from 1287 to 1314, with an average of 1300; GCLS, EBNA1: from 587 to 5100, with an average of 2007) and BAMH1-W (ADK, BAMH1-W: from 988 to 1202, with an average of 1095; GCLS, BAMH1-W: from 466 to 6460 with an average of 2627) copies for µl. Interestingly, both EBV genes were consistently detected in all technical triplicates of 8/26 (31%) ADK and 6/7 (86%) GCLS cases classified as EBER negative GC cases. Specifically, ADK showed from 0.9 to 1.5 copies/µl for EBNA1 and between 0.3 and 9.8 copies/µl cells for BAMH1-W whereas GCLS showed EBNA1 at low copy numbers ranging from 0.17 to 2.03 copies/µl for EBNA1 and between 0.46 and 41.85 copies/µl cells for BAMH1-W. Conversely, neither EBV gene was detected in any of the samples used as negative control (Table 2).

**Table 2:** Detection of EBV genome by applying conventional (EBER-ISH) and unconventional methods (ddPCR, RNAscope) in two different histological types of GC (ADK, GCLS)

Case Number		Assay			Histological Type
	EBER-ISH	ddPCR		RNAScope	
	EBERs	BamHI (copies/ul)	EBNA1 (copies/ul)	EBNA1 mRNAs (score)	
#1	−	2	0	–	ADK
#2	−	0	0	–	
#3	+	988	1287	2500	
#4	−	0	0	–	
#5	−	0	0	–	
#6	−	0	0	–	
#7	−	0	0	–	
#8	−	0	0	–	
#9	−	0	0	–	
#10	−	0	0	–	
#11	−	2.2	0	–	
#12	−	9.8	1.5	8	
#13	−	0	0	–	
#14	−	0	0	–	
#15	−	1.4	0	6	
#16	−	0.3	0	–	
#17	−	0.5	0	–	
#18	−	0	0	–	
#19	−	0	0	–	
#20	−	0	0	–	
#21	−	1.5	0.9	8	
#22	−	0	0	–	
#23	−	0	0	–	
#24	−	5.7	0	7	
#25	−	0	0	–	
#26	−	0	0	–	
#27	−	0	0	–	
#28	+	1202	1314	2600	
HCL		0	0		
HCL		0	0		
HCL		0	0		
#29	−	0.52	0.17	–	GCLS
#30	−	41.85	4	10	
#31	−	19.1	2.03	8	
#32	−	3.23	0.53	12	
#33	−	0.94	0.48	–	
#34	−	0.46	0.18	–	
#35	−	0	0	–	
#36	+	6460	2847	3000	
#37	+	1410	5100	2900	
#38	+	2710	1351	np	
#39	+	466	152	np	
#40	+	2089	587	np	
HCL		0	0		
HCL		0	0		
HCL		0	0		

### RNAscope and Immunofluorescence assays for the detection of EBNA1 mRNA in pan-keratin positive cells

**Fig. 2:**
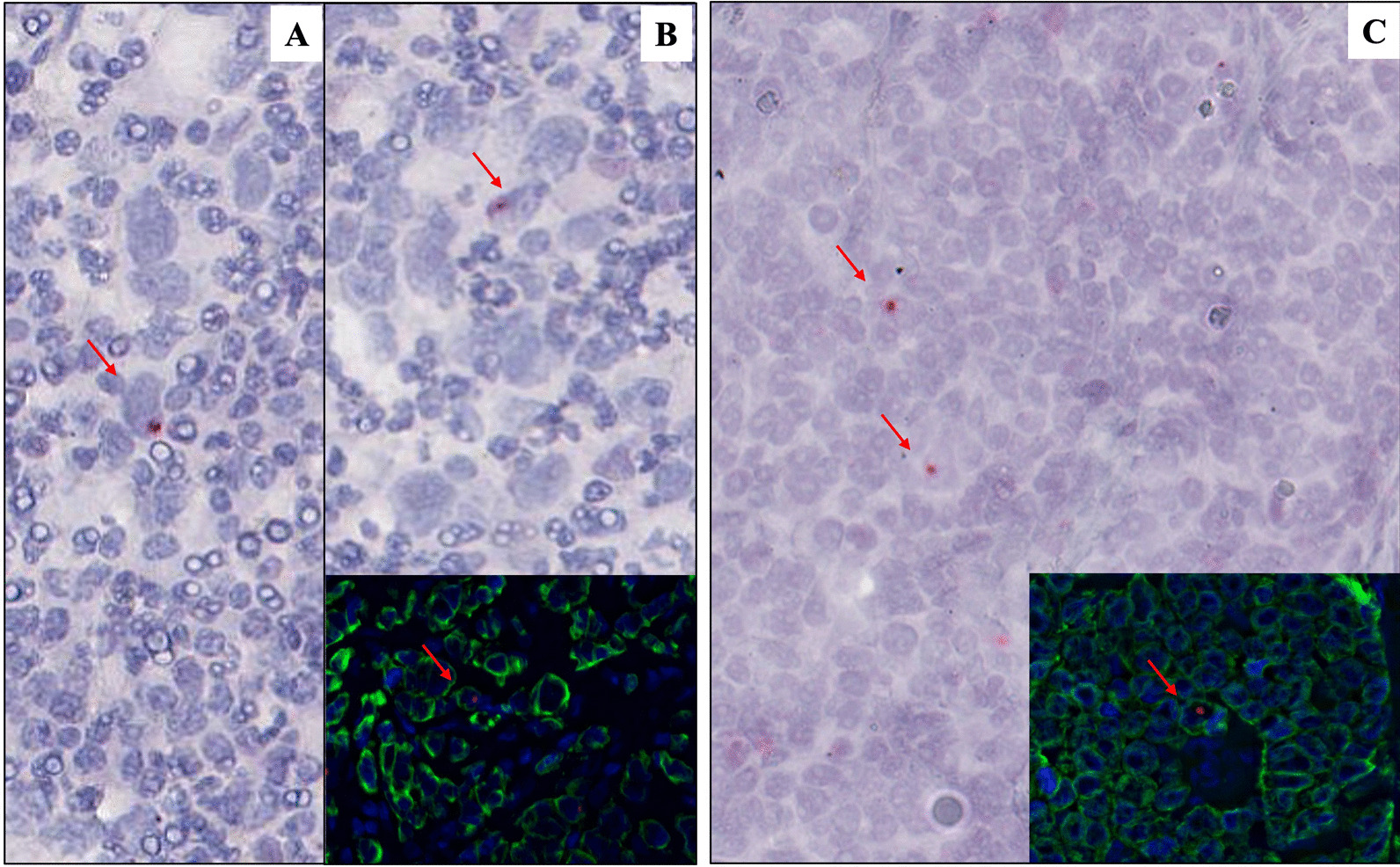
Sensitive in situ detection of EBV by RNAscope assay and immunofluorescence: Single red dot, produced by EBNA1 mRNA probes, were detected in EBER-ISH negative cases (**A**–**B**: GCLS; **C**: ADK). Double staining pan-keratin (IF, green) and EBNA1 (RNAScope, red) showed the presence of EBV traces in GC neoplastic cells (inset). Original magnification: 40X

To identify the morphological nature of cells contributing to the positive signals in ddPCR, we performed an in situ EBNA1 and EBER1 detection by applying RNAscope assay. The RNAscope for EBNA1 consistently documented the presence of multiple red dots in the nuclei of neoplastic cells of all EBER-ISH positive GC cases (ADK: from 2500 to 2600, with an average of 2550; GCLS: from 2900 to 3000, with an average of 2950) (Table 2). On the other hand, single red dots were only found in scattered rare cells of 4/26 (15%) ADK and of 3/7 (43%) GCLS cases classified as EBER-ISH negative but EBV positive by ddPCR (ADK: from 6 to 8, with an average of 7; GCLS: from 8 to 12, with an average of 10) (Fig. 2A–C; Table 2). Otherwise, all EBER-ISH negative/ddPCR negative samples did not show any EBNA1 signals (data not shown) while showing positivity for the PPIB control gene (data not shown). Finally, our results with RNAse preatreatment demonstrate a lack of signal after digestion (data not shown). To further confirm the neoplastic nature of EBNA1 positive cells, the EBNA1 mRNA positive sections were re-stained for pan-keratin immunofluorescent marker for the detection of GC tumour cells. A strong pan-keratin expression (green staining) was detected in the vast majority of cells of all GC cases while only scattered pan-keratin positive cells displayed a red punctate signal of EBNA1 mRNA (Fig. 2B, C, Inset).

Furthermore, our samples were tested with RNAScope assay for EBER1. The EBER-ISH positive cases showed a strong staining (data not shown), while it was negative in the cases with only traces of EBNA1 and BamHI-W by ddPCR (data not shown). Conversely, all EBER-ISH negative/ddPCR negative samples did not show any EBER1 signals (data not shown).

### Methylation assay findings

**Fig. 3:**
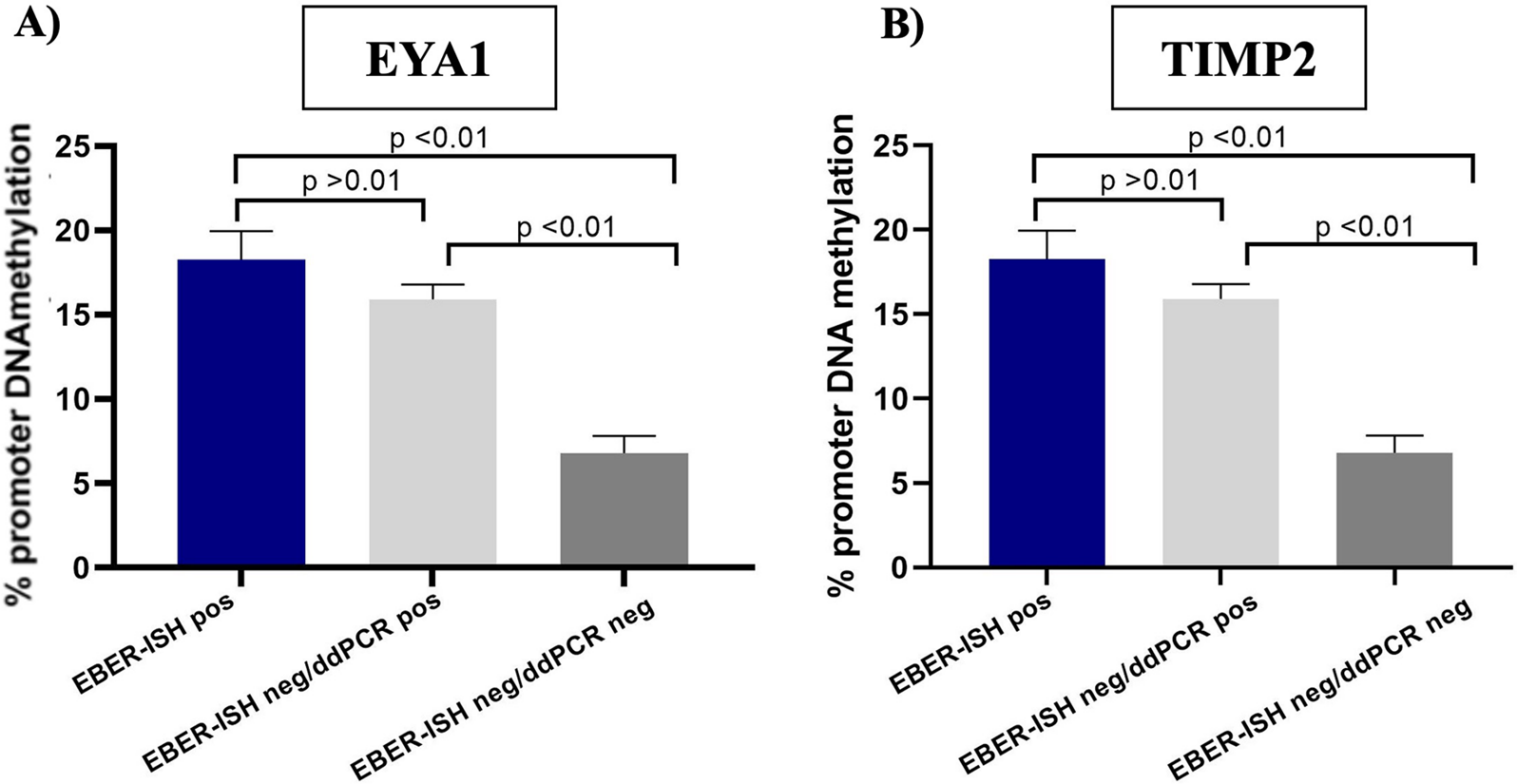
Methylation assay findings: The methylation level of *Timp2* (**B**) and *Eya1* (**A**) in 7 GC EBER-positive cases overlapped that detected in 14 GC EBERnegative/ddPCR-positive cases, whereas it was higher than observed in EBER-negative/ddPCR-negative cases. P values were calculated according to T-test

The methylation status of *Eya1* and *Timp2* genes in 7 EBER-ISH positive cases (2ADK, 5 GCLS) was compared to that observed in specimens classified as EBER-ISH negative/ddPCR-positive cases or EBER-ISH negative/ddPCR-negative. *Timp2* and *Eya1* presented a quite similar methylation pattern in both EBER-ISH positive and EBER-ISH negative/ddPCR-positive groups (18 and 16.5% respectively for *Timp2*; 18.25% and 15.9% for *Eya1*) and the lowest level of methylation in the EBER- negative/ddPCR-negative group (6% *Timp2*; 6.8% *Eya1*) (Fig. 3). *P* values were calculated according to T-test.

### Survival analysis

**Fig. 4:**
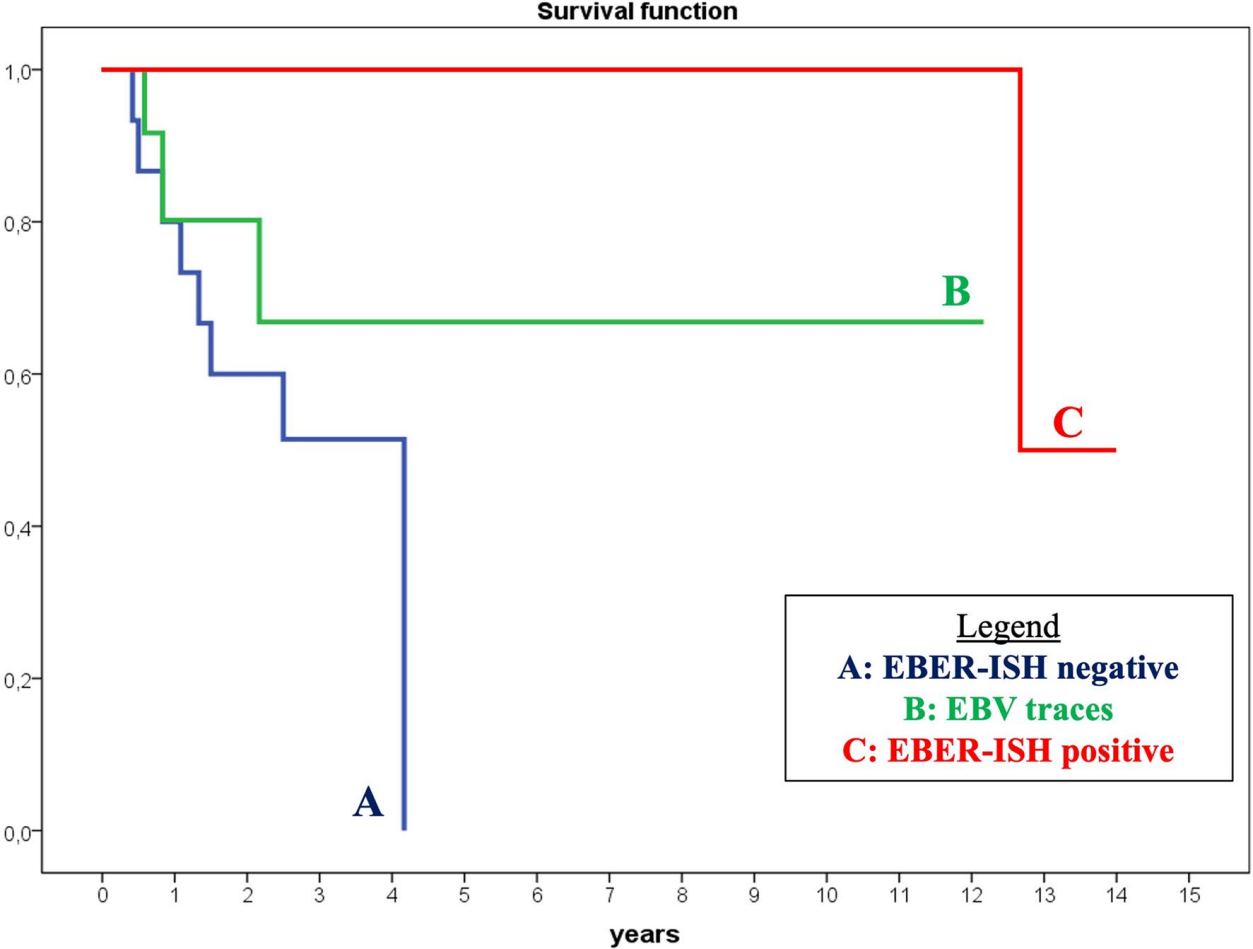
Kaplan–Meier curves of overall survival (OS) in the different patient groups. Kaplan–Meier curve showed a significantly better OS in EBER-ISH positive cases (**C**) than EBER-ISH negative cases (**A**). Although not significant, a notably difference was also found in cases with EBV traces (**B**) than EBER-ISH negative (**A**)

EBVaGCs are reported to be correlated with lower T and N stages, outlining a better prognosis than the EBER-ISH negative cases, especially during the early stage in the submucosa [38]. However, these findings are contradicted by other papers which highlight no differences in survival between EBVaGC and EBV-negative gastric carcinoma (EBVnGC) cases after surgery and/or conventional chemotherapy [39]. In our series, Kaplan–Meier curves showed that EBER-ISH positive cases have significantly better overall survival (OS) than EBER-ISH negative cases. Although not significant, a notable difference was also found in cases with EBV traces than EBER-ISH negative (Fig. 4). In our series, univariate Cox survival analysis showed that only viral state (including EBER-ISH negative, EBV traces and EBER-ISH positive) and T-stage were significantly correlated (*p* = 0.05) with OS (Tables 3, 4). However, multivariate analysis did not show significant prognostic independence between viral state and T-stage (*p* > 0.05) and no significant multivariate model could be identified.

**Table 3:** Mean survival time for each viral state (including EBER-ISH negative, EBV traces, EBER-ISH positive cases)

Viral state	Survival time (years)		
	Mean	95% Confidence interval	
		Lower bound	Upper bound
EBER-ISH negative	2.73	1.89	3.58
EBV traces	8.57	5.15	11.99
EBER-ISH positive	13.33	12.41	14.26
Global	8.02	5.21	10.83

**Table 4:** Univariate statistical association with different clinicopathological features, assessed by Cox survival analysis

Variable	*p* value
Age	0.96
Gender	0.63
Istotype Lauren classification	0.94
Grade	0.48
Viral state	0.05
T	0.05
N	0.74
M	0.12
STADIO	0.10

## Discussion

Through this study, we demonstrated the presence of EBV traces in EBER-ISH negative GC cases by applying highly sensitive methods for the viral genome detection, such as ddPCR and RNAscope. In particular, ddPCR showed the presence of BAMH1-W and/or EBN.

A1 segment regions in 8/26 ADK and in 6/7GCLS cell populations isolated through microdissection from conventional EBV-negative biopsies. ddPCR represents a robust tool able to essentially detect a single DNA template sequestered into a droplet containing even only one copy of the template target DNA. This approach allowed us to overcome the higher quantification variability of BamHI-W as reported by Sanosyan et al. [40]. Traces of a previous EBV infection were also confirmed and visualized by RNAscope for EBNA1 mRNA in 4/26 ADK and in 3/7 GCLS. Interestingly, the RNAscope staining often consisted of a single dot per cell in contrast to the more abundant EBNA1 labelling of infected EBER-positive GC cells that can be occasionally observed in tissue biopsies. Furthermore, by combing the RNAscope staining and IF detecting pankeratin marker, we confirmed the neoplastic nature of EBNA1 mRNA positive cells. These methodologies, directly assessing the presence of the virus in conventional EBV-negative primary tumours, suggest that EBV infection might have happened in the early pathogenesis of a significantly greater proportion of GC cases than commonly thought, in keeping with the *hit-and-run* hypothesis [15, 41]. This theory postulates that the transforming events initially provided by the virus are later functionally replaced by stable (epi) genetic changes of the host cell. Meanwhile, the acquisition of somatic mutations in cellular oncogenes\tumor suppressor genes will be functionally compensated for the loss of EBV genome. According to the hit and run hypothesis most of the EBV genome may be lost due to the imperfect duplication and asymmetric portioning of EBV episomes during S-phase and M-phase respectively. EBNA1 and BAMHI-W, which are the most conserved region of the viral genome, may be linearly integrated into the host genome and contribute to the traces detected by us, while other parts of EBV genome may be completely lost [36, 37, 42]. Indeed, our detection of EBNA1 and BAMHI-W, and not EBER, may be the result of such a phenomenon [43]. Numerous evidence suggests that the EBV genome can randomly integrate at vulnerable sites of the cellular genome resulting in host genome instability or deregulated gene expression [36, 44–46]. At the (epi)genetic level, EBVaGCs are quite different from EBVnGCs, presenting a gain-of-function in oncogenes and loss-of-function in tumour suppressor genes [18]. EBV infection has been described as a determinant factor of epigenetic alterations both in the viral and cellular genomes, suggesting a direct viral-mediated epigenetic modification with consequent EBVaGC tumourigenesis. The prevalence of DNA hypermethylation is reported to be significatively higher in EBVaGCs when compared to EBVnGCs. The EBV-induced epigenetic changes are heritable and not dependent on continued viral gene expression [14, 16, 34, 47]. Moreover, as they are retained after complete loss of the virus from the host cell as a mark of previous EBV infection, the methylation changes may provide a mechanistic framework for how the virus can act in a *hit-and-run* manner. Based on that, we studied the methylation status of genes reported to be extensively higher methylated in EBVaGCs than EBVnGCs such as *Timp2* and *Eya1* [29]. Our results showed that *Timp2* exhibited in GC harbouring only traces of EBV infection, a considerable level of methylation similar to that observed in conventional EBER-ISH positive cases and greater than in cases not featuring any traces of EBV infection, thus confirming the tight association between EBV infection and *Timp2* promoter methylation. A similar methylation pattern was also demonstrated for *Eya1*. Although the *hit-and-run* hypothesis is difficult to formally prove and our results are not a direct proof of it, altogether our findings are in accordance with the concept that EBV can be largely lost from the tumour cells and leaves epigenetic vestiges as proof of its previous infection. Finally, multivariate analysis displayed that viral state and T stage are non-independent prognostic factors, suggesting that the viral state may influence the tumour size, possibly by inducing a tumours microenvironment (TME) not favourable for the tumour growth [48, 49]. However, due to the small sample size reported in the present paper, further studies are needed to confirm our results in a larger cohort of cases.

## Conclusion

In conclusion, although based on a small sample size, our findings expand EBV contribution to gastric pathogenesis more widely than currently acknowledged and indicate the methylation changes as a mechanistic framework for how EBV can act in a *hit-and-run* manner. Finally, we found that the viral state was of prognostic significance in univariate and multivariate analyses, supporting the efforts toward the development of prophylactic vaccination strategies against this virus.

## Data Availability

All data are available from the corresponding author.

## Abbreviations

GC: Gastric cancer
ADK: Adenocarcinoma
GCLS: Gastric cancer with lymphoid stroma
ddPCR: Digital droplets PCR
EBV: Epstein–Barr virus
BL: Burkitt lymphoma
NPC: Nasopharyngeal carcinoma
LELC: Lymphoepithelioma-like gastric cancer
TCGA: The cancer genome atlas
EBVaGC: Epstein–Barr virus associated gastric cancer
MSI: Microsatellite instable tumours
GS: Genomically stable
CIN: Chromosomally instable
HL: Hodgkin lymphoma
FFPE: Formalin fixed paraffin embedded
WHO: World Health Organization
IF: Immunofluorescence
EBVnGC: Epstein–Barr Virus negative gastric cancer
TME: Tumor microenvirnment
EBER-ISH: Epstein–Barr virus (EBV)-encoded RNA (EBER)
EBNA1: Epstein–Barr nuclear antigen 1
OS: Overall survival
